# Complications after Thermal Ablation of Hepatocellular Carcinoma and Liver Metastases: Imaging Findings

**DOI:** 10.3390/diagnostics12051151

**Published:** 2022-05-05

**Authors:** Federica De Muzio, Carmen Cutolo, Federica Dell’Aversana, Francesca Grassi, Ludovica Ravo, Marilina Ferrante, Ginevra Danti, Federica Flammia, Igino Simonetti, Pierpaolo Palumbo, Federico Bruno, Luca Pierpaoli, Roberta Fusco, Andrea Giovagnoni, Vittorio Miele, Antonio Barile, Vincenza Granata

**Affiliations:** 1 Department of Medicine and Health Sciences V. Tiberio, University of Molise, 86100 Campobasso, Italy; demuziofederica@gmail.com; 2 Department of Medicine, Surgery and Dentistry, University of Salerno, 84084 Fisciano, Italy; carmencutolo@hotmail.it; 3 Division of Radiology, Università degli Studi della Campania Luigi Vanvitelli, 80138 Naples, Italy; federica.dellaversana@studenti.unicampania.it (F.D.); francescagrassi1996@gmail.com (F.G.); 4 Division of Radiology, Università degli Studi di Napoli Federico II, 80138 Naples, Italy; l.ravo@studenti.unina.it (L.R.); marilina.ferrante@gmail.com (M.F.); 5 Division of Radiodiagnostic, Azienda Ospedaliero-Universitaria Careggi, 50134 Firenze, Italy; ginevra.danti@gmail.com (G.D.); federicaflammia91@gmail.com (F.F.); vmiele@sirm.org (V.M.); 6 Italian Society of Medical and Interventional Radiology (SIRM), SIRM Foundation, Via della Signora 2, 20122 Milan, Italy; palumbopierpaolo89@gmail.com (P.P.); federico.bruno.1988@gmail.com (F.B.); antonio.barile@univaq.it (A.B.); 7 Radiology Division, Istituto Nazionale Tumori—IRCCS—Fondazione G. Pascale, Napoli, Italia, Via Mariano Semmola, 80131 Naples, Italy; igino.simonetti@istitutotumori.na.it (I.S.); v.granata@istitutotumori.na.it (V.G.); 8 Department of Diagnostic Imaging, Area of Cardiovascular and Interventional Imaging, Abruzzo Health Unit 1, 67100 L’Aquila, Italy; 9 Department of Applied Clinical Sciences and Biotechnology, University of L‘Aquila, 67100 L‘Aquila, Italy; 10 Departement of Radiological Sciences, Università Politecnica Delle Marche, University Hospital, Via Tronto 10, 60126 Ancona, Italy; l.pierpaoli@pm.univpm.it (L.P.); a.giovagnoni@univpm.it (A.G.); 11 Medical Oncology Division, Igea SpA, 80013 Naples, Italy

**Keywords:** RFA, MWA, complications, imaging, HCC, metastases

## Abstract

Tumour ablation is a strategy of treatment of hepatic tumours in patients with small hepatocellular carcinoma (HCC) (<3 cm) or in patients unfit for surgical resection. Moreover, tumor ablation can be used as an adjuvant therapy or may be used in association with resection in case of patients with poor functional liver disease. These types of treatment usually could be performed percutaneously under image guidance. The most clinically verified and used ablation modalities are Radiofrequency Ablation (RFA) and microwave ablation (MWA). However, despite both of them are considered minimally invasive techniques, they could be related to post-procedural complications. The International Working Group on Image-Guided Tumor and the Society of Interventional Radiology (SIR) identified major and minor post-ablative complications. Major complications, as vascular complications, occur in 2.2% to 3.1% of cases and include all the high risk pathological conditions which could increase the level of care or result in hospital admission or substantially prolonged hospital stay (SIR classifications C–E). Minor complications, as biliary complications, occur in 5% to 8.9% and include self-limiting conditions that are considered to be of low risk for the patient’s outcome. The purpose of this review is to summarise the main pathological ultrasound (US) and Computed Tomography (CT) findings, that may arise after ablative treatment. To simplify the analysis, the pathological pictures are divided according to the site of damage into vascular, biliary and extrahepatic complications.

## 1. Introduction

Ablation therapy is a widely used treatment strategy in liver diseases. The main indication guidelines are hepatocellular carcinoma (HCC) and metastatic lesions, in patients with small HCC (<3 cm) or ineligible for surgical resection [1,2,3,4]. In addition, tumour ablation can be applied as adjuvant therapy after surgery or in combination with tumour resection in patients with an impaired liver function [5]. The most clinically verified ablation treatments are Radiofrequency ablation (RFA) and microwave ablation (MWA), both of them apply thermal energy to heat tissues to at last of 60 °C for maximum efficacy [6]. Although the technical features of these percutaneous procedures are similar, they differ for physical phenomenon on the basis of heat generation [5]. Specifically, RFA causes cellular death thanks to thermocoagulation necrosis, while MWA used a dielectric heating [7]. The MWA has some advantages over RFA, such as greater volume of cellular necrosis (about 5 cm of target compared to 3 cm), procedure time reduction (about to 20 min) and higher temperatures delivered to the malignant tissue (according to manufacture utilized) [5,7]. Even more important, MWA shows less susceptibility to variation in the morphology of the ablated zone because of heat-sink effects from adjacent vasculature [5,7]. The main feature that should be considered during the indication of percutaneous treatments is the possibility to ablate all viable tumour tissue and achieve an adequate tumour-free margin [5,8,9,10,11]. As regards the treatment of HCC, ablative procedures are recommended when a maximum of three lesions have to be treated (specifically a single lesion smaller than 5 cm or as many as three nodules smaller than 3 cm) [5], while in the treatment of colorectal cancer metastases the number of lesions is not strictly a contraindicating factor [12,13,14,15]. Although ablation procedures are considered safe in most cases, several complications related to mechanical or thermal damage may be observed at follow-up examination. Hence radiologists have not only to assess tumour response, but should become familiar with the main radiological features that occur after the procedure [16,17,18]. The International Working Group on Image-Guided Tumour and the Society of Interventional Radiology (SIR) identified major and minor post-ablative complications [19,20]. Major complications occur in 2.2 to 3.1% of cases and include all the high risk pathological conditions which could increase the level of care or result in hospital admission or substantially prolonged hospital stay (SIR classifications C–E) [19,20]. Minor complications occur in 5% to 8.9% and include self-limiting conditions that are considered to be of low risk for the patient’s outcome [19,20]. Complications are also distinguished according to the time of insurgence into immediate (6–24 h after the procedure), periprocedural (within 30 days) and late injuries (after 30 days) [19,20]. Different imaging techniques could be applied in post-procedural follow-up [21,22], but Computed Tomography (CT) is definitely the gold standard method. The spatial resolution and the possibility to perform multiplanar reconstructions (MPR) make CT ideal for an extensive analysis of the resection margin [23,24,25,26,27,28]. In addition, fast thoraco-abdominal scans enable to an early detection of possible complications, allowing an assessment of their extent and severity. On the other hand, CT is burdened by radiation exposure. Considering that short-term post ablative surveillance is performed at close intervals, Ultrasound (US) even with contrast medium injection can be considered as an excellent alternative [29,30,31,32,33,34,35,36,37]. In the last few decades, contrast-enhanced ultrasound (CEUS) has assumed an increasingly recognised role not only in guiding ablative procedures but also in identifying possible complications, especially haemorrhagic ones [20,38]. The purpose of this review was to summarise the main pathological US and CT findings, that may arise after ablative treatment. To simplify the analysis, the pathological pictures were divided into vascular, biliary, infectious and extrahepatic complications. 

## 2. Vascular Complications

### 2.1. Bleeding

**Figure 1:**
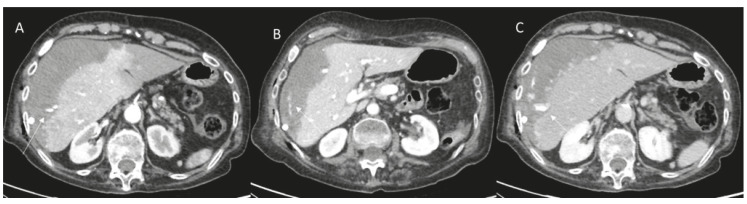
Woman 65 year at 7-day follow-up after RFA of liver metastases. CT assessment ((**A**): arterial phase; (**B**): portal phase and (**C**): late phase): active bleeding (arrow).

Intraperitoneal bleeding is the most frequent major complication (Figure 1), that can be immediate/periprocedural/late injury, occurring in 0.1–0.4% [5].

Usually, the direct mechanical injury, rather than thermal damage, is the cause of blood spillage.

The cirrhotic patients are considered on high-risk, due to the underlying coagulopathy caused by hepatic hypofunction.

When large-calibre electrodes (17–14 gauge) are used during the procedure, it is appropriate to set a biochemical coagulation panel, with the possibility of postponing the treatment in case of altered values [39]. 

The use of multiple electrodes and treating lesions located next to large vessels increase the likelihood of bleeding occurring.

To avoid this adverse event, operators should leave a large margin of healthy parenchyma in the route of the electrodes, being careful not to cross vessels which could therefore be damaged.

Continuous monitoring the procedure with Colour Doppler US, may lead to find a source of bleeding through observation of a localized swirling or turbulent flow [40].

CEUS appears to be more sensitive in identifying a haemorrhagic spillage, overcoming respiratory motion artefacts, which may affect Colour Doppler performance.

Specifically, applying a high-powered flash during a continuous acquisition, the examiner could disrupt the contrast microbubbles within the volume and temporarily reset perfusion. In this way, a continuous extravasation of contrast medium due to ongoing haemorrhage could be intercepted [41].

Also, CEUS seems also to be capable to classify more severe patients, who need immediate surgery [42].

Indeed, in patients with suspected abdominal haemorrhage two main US patterns could be observed: an oval hyperechogenic blush associated with minor blood extravasation and a high-flow fountain-like contrast spillage due to a potentially lethal hemorrhage [42].

Although US demonstrates good diagnostic accuracy, in emergency setting [43,44,45,46,47,48,49], a periprocedural haemorrhage should then be investigated also through a multiphasic CT study [50,51].

On unenhanced scan haemorrhage is recognisable as a highly attenuated fluid collection surrounding the liver parenchyma or in peritoneal space.

During the arterial phase, active bleeding is visible as a clear overflow of iodinated contrast agent, which could assume a jet-like appearance, evocative of arterial high-flow bleeding.

Within 24 h after the procedure, the blood could organise into a hematoma. On basal CT scan hematoma appears as a biconvex or growing intraparenchymal area with a suprafluid density value (between 50 and 60 HU), usually superficial at the entry point of the electrode.

Suspected hypoechoic/hypodense areas detected on baseline US and CT scan could be diagnosed as hematomas after contrast injection, showing microcirculation alterations within the collection in absence of contrast enhancement [52].

### 2.2. Vascular Thromboses

Besides bleeding, hepatic vessels thrombosis is another frequent vascular complication that usually could occur few hours after the procedure. According to the vessel involved it can be a major or minor complication.

The vascular damage caused by the heat-sink effect depends on the calibre of the vessel and the blood flow rate. Thus, it tends to affect vessels of reduced calibre, with a slowed down flow due to occlusions for former treatment or pre-existing thrombosis [53]. 

Other risk factors to consider are a central location of the tumour, veins compression and mechanical direct damage induced by the electrodes placement.

On US images a portal or hepatic vein thrombus appears as a circumscribed, intraluminal hyperechogenic spot in absence of inner flow signs on Color Doppler US.

On contrast enhanced (CE)-CT study, a portal intraluminal filling defect may be best depict on arterial phase, frequently accompanied by a segmental enhancement of the adjacent liver parenchyma, paradoxally of increased attenuation due to a compensatory augmentation of local arterial flow [54].

On the other hand, a thrombosis of the hepatic veins is best recognised in the portal or equilibrium phase, often associated with a parenchymal wedge area of reduced enhancement driven by vascular congestion [55].

In this last circumstance, pervious portal branches could be distinguished at the margins of the wedge area, allowing a certain differentiation between portal ischemia and hepatic venous congestion [40].

Extensive portal thrombosis may lead to parenchymal infarction, recognisable as a triangular-shaped portion of unenhanced liver parenchyma during arterial phase on CECT and CEUS examination [56]. 

The infarcted area spreads over the hepatic surface, in the context of which portal segments with air attenuation are often visible. 

The lesion may extend on an entire lobe, causing liver failure. However, in most cases the infarction is symptomless and tends to dissolve over time, only applying antibiotic therapy [52].

### 2.3. Pseudoaneurysm

During the arterial phase of a CECT study, the radiologist should pay attention to the possible presence of a small, well-circumscribed hyperdense nodule within the ablated area. This finding could suggest a small pseudoanerismatic sac, due to iatrogenic damage of a branch of the hepatic artery.

On US examination, pseudoaneurysm could be seen as an anechogenic zone similar to a cyst formation.

Hence, the diagnosis could be easily achieved using Colour Doppler imaging, through the characteristic *yin-yang sign* due to the turbulent flow within the lesion [57].

Pseudo-aneurysms, its self a minor complication, should be monitored over time, given the possible rupture with an inauspicious outcome, becoming a major complication. The evidence of progressive diameter growth in subsequent follow-ups, may determine the need of an interventional procedure (i.e., embolization) [58].

## 3. Biliary Complications

### 3.1. Ductal Stenosis and Biloma

**Figure 2:**
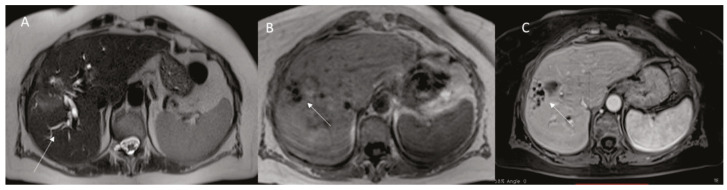
Woman 63 year at 1-month follow-up after RFA of liver metastasis. MRI assessment ((**A**): HASTE T2-W sequence; (**B**): in phase T1-W sequence and (**C**): porta phase of contrast study), ablated zone with biliary tree damage (arrow).

Biliary ducts thermal injuries or stenosis (Figure 2) are common post-ablative minor complications, particularly involving peripheral branches which are more susceptible to heat-induced damage. The incidence reported vary from 0% to 10.5%, according to the employed technology and lesions type [5], although, probably, the asymptomatic cases have not been reported.

If the operator overcomes the heat sink effect of large-calibre vessels adjacent to a central mass, also a damage to the main bile ducts may be determined. Therefore, the ratio of injury risk and therapeutic benefit has to be estimated for each patient. 

In cases that are more complicated a combination therapy with transarterial chemoembolization or percutaneous ethanol injection should be considered [39].

Biliary stenosis could evolve over months and does not require specific treatment as it tends to be subclinical, especially peripheral ones.

**Figure 3:**
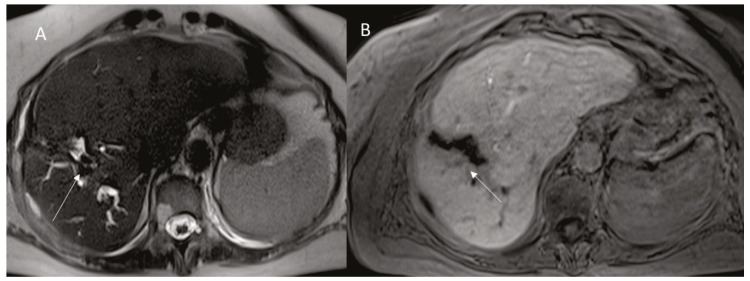
The same patient of Figure 2, MRI assessment ((**A**): HASTE T2-W sequence, (**B**): EOB-Phase of contrast study) after 2-month, arrow shows biloma.

However, biliary ducts necrosis due to the pre-stenotic dilatation may lead to the leakage of bile, collecting in intrahepatic (Figure 3) or extrahepatic biloma.

US imaging identifies an oval or round cyst formation, which could show a simple fluid content or a mixed collection of cellular debris and blood clots. A heavily multiloculated structure are usually associated with infected bilomas.

In most cases of CT examinations is required to allow a conclusive diagnosis [59].

Usually, on CECT a biloma appears as a hypodense fluid collection (<20 HU) often with a fibrotic capsule, formed thought the mild bile-induced inflammation process of the adjacent liver parenchyma.

In addition to the classic findings, a characteristic CT *mural nodule in cyst* pattern of mixed content bilomas could be appreciated after an ablative procedure. 

The *mural nodule* represents residuals necrotic tumor debris with a higher density than the surrounding bile, lacking of enhancement after contrast medium injection [60].

**Figure 4:**
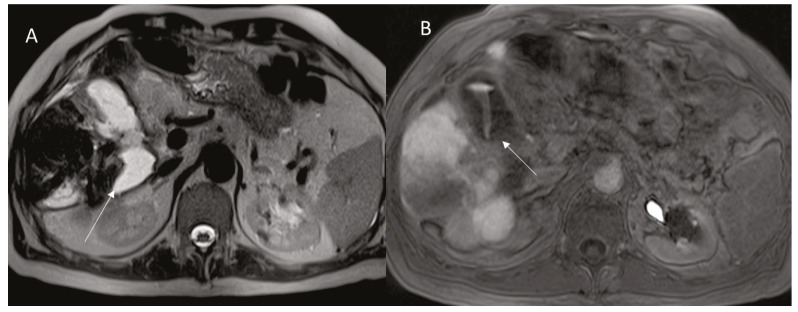
Man 74 year at 1-month follow-up after RFA of HCC on VI seg. MRI ((**A**): HASTE T2-W sequences in axial, in (**B**): EOB-phase of contrast study in axial plane). The arrow shows bile leak.

Biloma formation occurs in a small percentage of cases after ablative procedures. Chang et al. in a study on HCC observed the development of bilomas in 3.3% of all sessions of RFA and only one of the 109 examined collections required drainage as it was overinfected [61] or for biliry leak (Figure 4).

However, catheter drainage and antibiotics should be promptly employed once biloma is diagnosed to avoid uncontrolled spread of the infection, reducing rates of morbidity and mortality.

### 3.2. Hemobilia

Ablation procedures could be an iatrogenic cause of blood extravasation into the bile ducts, with an incidence of 0.25% as reported by Rhim et al. [39].

Goto et al. [62] found an increased hemobilia occurrence in the case of RFA ablations conducted on caudate lobe in the deeper portions of the liver, due to a risk of damaging both vessels and bile ducts during the procedure [62].

The detection of clots, especially small ones, inside bile ducts on US examination could be difficult due to their loss of ecogenicity and shape, leaning against the ductal walls [63].

The US contrast medium injection could play an important role in this situation.

In fact, Francica et al. [64] found different pathological signs at CEUS, surely related to active or inactive status of the hemobilia. Examiners found, within the lumen of the gallbladder, contrast microbubbles in active blood spillage and hyperechogenic material referable to blood clots with no enhancement in a hemobilia that spontaneously stopped [64].

The presence of scattered hyperdensities within bile ducts are signs of haemobilia at basal CT scan.

Moreover, the enhancement of the ductal walls after contrast injection may indicate a cholangitic process triggered by the continuous haemorrhagic stimulus on the ductal walls [65].

Clinically patients could manifest jaundice, melena and abdominal pain and treatment could range from supportive medical care to interventional radiologic or surgical intervention depending on the severity of the process.

### 3.3. Cholecystitis

**Figure 5:**
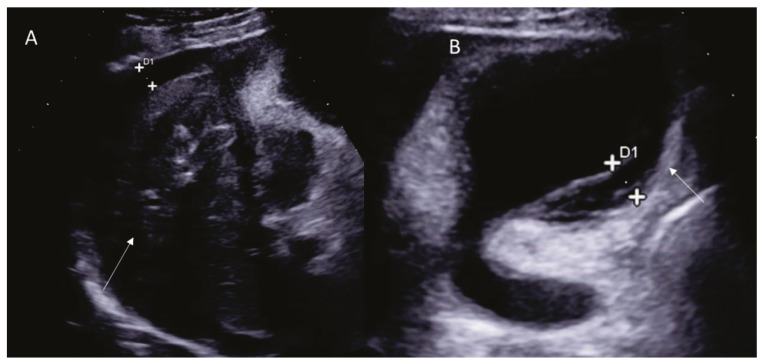
Woman 58 year at 1-week follow-up after MWA of liver metastasis. MRI assessment US assessment of RFA treated HCC on V segment ((**A**); arrow). In (**B**) arrow shows cholecystitis.

In treating masses adjacent to the cholecystic fossa, complications such as cholecystitis may occur (Figure 5).

Nevertheless, the most frequent pathological change observed after an ablation procedure is transient wall thickening of the gallbladder and acute symptomatic inflammation or perforation are considered rare events. 

This is probably due to protective effect of the lumen fluid component against thermal damage, which prevent the occurrence of potentially more serious injuries [66].

Regardless the gravity of the process, the imaging findings are easily recognisable to an expert eye. 

Parietal thickening associated with pericholecystic fat stranding should lead to the suspicion of an acute cholecystitis, which may be further complicated by peritoneal infection.

Moreover, the creation of a fistula between a vessel and the intrahepatic bile ducts, or thermal necrosis of the bile ducts, could lead to a haemocolecyst. 

In this case, both CT and US may detect echogenic/homogeneously material forming a pseudomass, within the lumen of the gallbladder [67].

Simple fluid collections may be drained percutaneously, while in case of a complicated inflammation, a laparoscopically cholecystectomy may be required [68].

## 4. Infectious Complications

### Abscess

Bacterial colonization of the ablated area could lead to an abscess formation [69,70,71,72,73,74,75,76,77], which is considered a major complication occurring in 0.3–2% of all procedures [58].

The main risk factors are the presence of a biliary-enteric anastomosis with a possible ascending pathogen progression, an external drainage, an incompetent Oddi sphincter, diabetes and retention of iodized oil from previous tumor arterial chemoembolization [78,79].

The symptoms (fever, malaise, chill, pain at the treatment site, nausea, and vomiting) could simulate a postablation syndrome, a condition that occurs 1 to 3 days after treatment with spontaneous resolution in 1–2 weeks.

However, in case of an abscess, fever tends to be of higher grade and symptoms appear several days after the procedure [52]. 

Imaging is certainly very supportive and an abscess could be easily recognised with both US examination and CT scan.

On US, lesion appearance depends on size and content, usually ranging from a well- or poorly defined hypoechogenic nodule to a large inhomogeneous mass with septa and cellular debris, in absence of central perfusion on Color doppler examination [80].

On CEUS abscesses appear as inhomogeneous enhancing lesions with a persistently hypoechoic core, a rim of dense opacification and thin vessels along the septa and walls [81]. 

On CT examination, the typical imaging feature is a central hypodense core of fluid material surrounded by a hyperdense rim and a hypodense outer ring as a *double target* appearance. 

The inner rim shows an early and persistent enhancement and represents the wall of the collection, while the outer rim corresponds to the area of edematous liver parenchyma that only enhances on delayed phase [82].

Pleomorphic airborne microbubbles are usually visible within the lesion, which may help in the definitive diagnosis. Nevertheless, it should be noted that the presence of small amount of air in the ablated area is a physiological finding in 63% of cases, which persists for up to one month after surgery [83].

Percutaneous abscess drainage should be considered in patients with a decline in physical condition and a worsening of laboratory parameters [84]. 

Prophylactic antibiotic therapy is a matter of debate, and only few centres agree to adopt this strategy to prevent the formation of infected collections.

## 5. Extrahepatic Complications

### Gastro Intestinal Tract Injuries

The gastrontestinal tract (GIT) injuries are mostly periprocedural complications that may deeply affect patients’ prognosis. 

Wall damage may occur in procedure performed on a lesion close to a bowel loop or in the presence of fibrotic adhesions from previous treatments with an altered anatomy. 

The incidence of bowel perforation after RF ablation has been reported between 0.1–0.3% [40].

The colon, in particular the hepatic flexure, is more susceptible to perforation due to its poor mobility and reduced wall thickness. 

In contrast, the thick gastric wall and small bowel peristaltic movements appear to be protective factors against iatrogenic insults [78]. 

Although rare, a direct injury could potentially occur in any site adjacent to the treated liver area, so radiologists should promptly recognise its early imaging signs.

In cases of suspected perforation CECT should be performed.

Jeong et al. observed in a group of 52 patients receiving ablative treatments, that wall thickening >1.65 cm and concentric bowel wall thickening with mucosal disruption were main CT signs of a serious bowel damage, requiring urgent surgical treatment [85]. 

In addition, the presence of extraluminal air around a bowel tract and abdominal free fluid are other CT findings, which may suggest a perforation.

To prevent wall injuries, artificial ascites could be a valuable strategy, allowing a detachment of the liver parenchyma from hepatic flexure. 

While, for lesions located in left hepatic segments, a mechanically displace of the stomach using a multi-needle technique or a simple and practical ingestion of cold water could be solutions to prevent gastric walls injury [86].

## 6. Thoracic Complications

**Figure 6:**
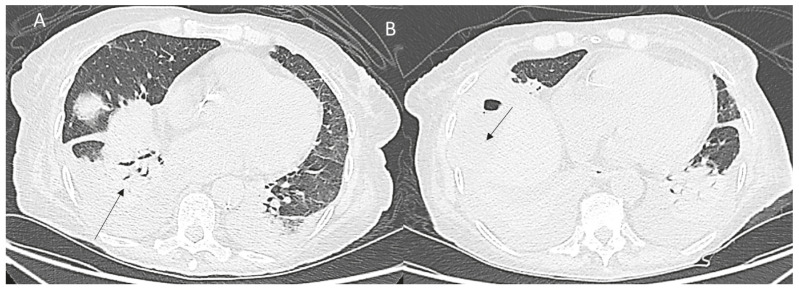
CT assessment (**A**,**B**) of treated HCC on IVa seg: pleural effusion and consolidation (arrow).

Thoracic complications may range from a right pleural effusion considered a paraphysiological sequela to severe pleural or diaphragmatic injuries [87] (Figure 6).

The risk of pleural damage increases during the treatment of masses located on the hepatic dome, choosing an intercostal approach. 

If the patient complains dyspnea and breathing difficulties, it is appropriate to perform a chest X-ray, eventually followed by a CT scan.

A pneumo or haemothorax due to damage of diaphragmatic vessels may be easily detected and subsequently monitored or eventually treated. 

Thermal diaphragmatic damage is another rare thoracic complication of liver ablation [88].

Although generally it’s a self-limiting condition, clinicians should be aware of this adverse event, which could be associated to bowel herniation and subsequent perforation.

## 7. Tumor Seeding

Recent large series studies reported the incidence of tumor seeding around 0.2–0.9% [40].

Post-procedural CT could successfully intercept a iatrogenic dissemination of disease.

On CT, tumour nests appear as enhancing, irregular soft tissue nodules along the ablation track similar to that of the primary tumor, not to be confused with accessory splenic nuclei or peritoneal plenosis. 

The risk of malignant dissemination increases with the number of electrode repositioning, the treatment of superficial or subcapsular tumor and obviously with a poor tumor differentiation and aggressive tumor histology [89].

An adequate heating of the ablation track is essential to prevent secondary tumor diffusion, which is the most feared long-term complication.

## 8. Fistulae

The imaging findings of an abscess and pneumobilia should always give rise to the suspicion of a fistula formation.

Depending on the location of the tumour, various ablation-related fistulous pathways may form.

In treating lesions on hepatic dome, damaging of the biliary duct and pleura may result in a biliary-pleural fistula. The risk is higher when lesions are located within segments VII and VIII [88].

Despite pleural involvement, Thiemann et al. also reported the possible development of a hepato-pericardial fistula leading to pericardial empyema after RFA procedure on metastatic hepatic lesions [90].

In all these circumstances, CT is the imaging method of choice, enabling to detect the direct passage of contrast medium agent through the fistulous path.

As previously reported [5], the operator’s experience and manual dexterity are essential prerequisites for avoiding this major complication, which can potentially require extensive surgery [5].

Furthermore, it is essential to carefully select the patients who could undergo to an ablation procedure, always bearing in mind the risks and complications which may follow [91].

Hence, during the preoperative setting it should be also considered electroporation-based treatments, i.e., electrochemotherapy (ECT) and irreversible electroporation (IRE) [92,93,94,95,96,97,98,99,100], especially in the treatment of large central tumors near to the main bile ducts or major vessels [101,102,103]. 

## 9. Conclusions

The knowledge of post ablation complications radiological findings can be helpful to detect immediately the main complications that can arise after an ablative procedure, allowing the possibility of early and specific treatment. CT with multiphase contrast study remains the tool to choose in emergency setting, while CEUS is the diagnostic tool that could be used during treatment and as a surveillance tool. MRI with hepatospecific contrast could be used in selected cases (as biliary leak).
